# Epidemiology of Activated Protein C Resistance and Factor V Leiden Mutation in the Mediterranean Region

**DOI:** 10.4084/MJHID.2011.037

**Published:** 2011-09-08

**Authors:** Mehrez M. Jadaon

**Affiliations:** Department of Medical Laboratory Sciences, Faculty of Allied Health Sciences, Kuwait University, Kuwait

## Abstract

Venous thromboembolic disorders (VTE) are serious disorders with high morbidity and mortality rates. Many genetic and acquired risk factors were identified to cause VTE. The most common genetic risk factor is Factor V Leiden mutation (FVL). FVL was found in high percentage of populations of Caucasian origin but was almost absent in non-Caucasians. It was also reported in populations living in North Africa and the Middle East. This review article briefly explains FVL and how it causes VTE, the distribution of FVL worldwide, and then it elaborates on the epidemiology of FVL in the Mediterranean Region and how this brought speculations that FVL might have originated in the Eastern Mediterranean area.

## Introduction:

Thrombophilia is the term given to abnormal blood coagulation condition leading to increased tendency towards coagulation (hypercoagulability status). People with hypercoagulability are at risk of developing thrombosis, especially venous thromboembolic disorders (VTE) including deep vein thrombosis (DVT) and pulmonary embolism (PE). VTE is a significant cause of morbidity and mortality in many countries with an annual incidence of 1/1000.^1^–^4^ Many genetic and acquired risk factors for the development of VTE were identified. In fact, the WHO expert group (1996) defined thrombophilia as a tendency to develop VTE that may be genetically determined, acquired or both.^5^ Genetic factors include activated protein C resistance (APC-R) associated with Factor V Leiden mutation (FVL), Prothrombin G20210A mutation associated with high levels of prothrombin, genetic deficiencies of proteins C, S and antithrombin, and others. Acquired risk factors include lupus anticoagulants, pregnancy, use of contraceptives, major surgeries, cancer, inflammations, and others. This review article focuses on the epidemiology of APC-R/FVL in the Mediterranean area. For a better understanding of the pathophysiology involved in causing hypercoagulability by APC-R/FVL, it may be helpful to start by a quick revision of the process of coagulation and its associated natural anticoagulants.

## Blood Coagulation:

**Figure 1.**
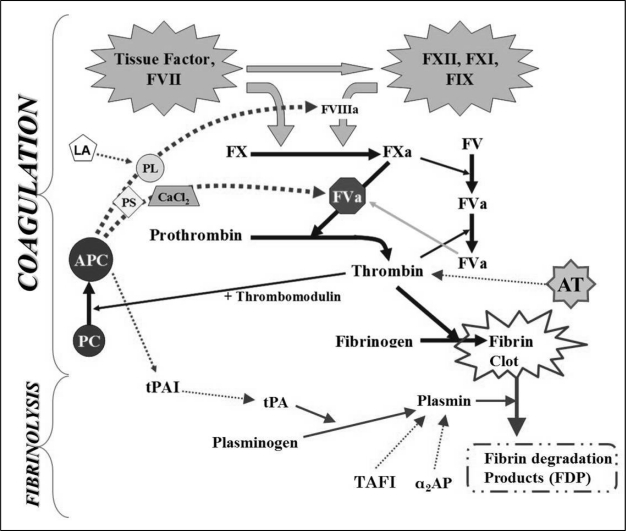
The processes of coagulation and fibrinolysis as a series of chemical reactions leading to the formation of a clot to stop bleeding from the site of injury, and then removing the clot afterwards. Solid lines indicate activation process, while dashed lines indicate inactivation process. Abbreviations: antithrombin (AT), protein C (PC), activated protein C (APC), protein S (PS), phospholipids (PL), lupus anticoagulants (LA), tissue plasminogen activator (tPA), tPA inhibitor (tPAI), α2 antiplasmin (α2AP), thrombin activatable fibrinolysis inhibitor (TAFI).

Normally, blood loss through injured vessels is prevented by a normal physiological process called “Hemostasis”. Normal human hemostasis is a balanced system which, on one hand, prevents excessive bleeding from any injured site, while on the other hand maintains blood circulation inside intact blood vessels by inhibiting intravascular coagulation. An efficient hemostatic process possesses intrinsic well-balanced regulatory systems, involving a number of dynamic mechanisms and chemical and physical reactions. It usually includes platelets, blood vessels and the coagulation system. In blood coagulation, a prominent response to an injury is recruited in the form of series of stepwise (cascade) chemical interactions leading to fibrin formation. This complex system involves certain proteins called the plasma clotting factors (enzymes). These enzymes circulate in the blood in an inactive form, and get activated in case of vessel injury. In summary, when a blood vessel is injured, the coagulation cascade is initiated by the release of tissue factor (thromboplastin) and the exposure of intravascular collagen, which activates clotting factors VII and XII, respectively. These clotting factors activate other clotting factors in a stepwise procedure ending up with the formation of a fibrin clot. A fibrin clot, in association with platelets, form a plug that blocks the injured blood vessel, preventing bleeding and allowing for wound healing. After healing, the fibrin clot is dissolved by the enzyme plasmin in a process called fibrinolysis. The whole process is under careful supervision by three main proteins that circulate normally in the blood; namely protein C (and its active form activated protein C; APC), protein S (PS) and antithrombin (AT). These so-called “natural anticoagulants” monitor the processes of coagulation and fibrinolysis in order to prevent excessive clotting.^6^,^7^,^8^ Abnormalities in clotting factors may lead to bleeding problems (hemophilia), while abnormalities in the natural anticoagulants may lead to hypercoagulability and thrombosis, with certain exceptions in both. Figure 1 gives an illustrative drawing of the process of coagulation and its associated fibrinolysis process and natural anticoagulants.

## Activated Protein C Resistance and Factor V Leiden Mutation:

**Figure 2.**
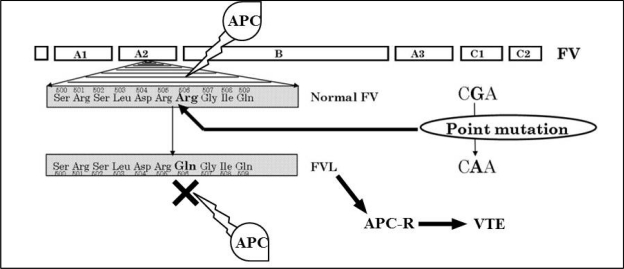
FV molecule showing arginine 506 as a main point of action for APC which is negatively affected by FVL.

In 1993, a Swedish research team led by B. Dahlbäck recognized an unusual phenomenon affecting the coagulation system. They were studying the effect of addition of external APC to plasma of patients with VTE. Normally, APC should inactivate clotting Factor V (FV) (Figure 1) and therefore slow down the coagulation process. However, in certain patients studied by Dahlbäck and his team, this slowing down did not occur. They called this phenomenon “APC resistance”, and they originally though this could be due a deficiency in a yet unknown protein that co-helps APC in inactivating FV.^9^ One year later, another group of researchers from Holland, led by R. M. Bertina, discovered a missense point mutation in the FV gene, where adenine (A) replaced guanine (G) at nucleotide position 1691 of exon 10 of the FV gene, only eleven nucleotides upstream of the beginning of intron 10. They called this mutation as FV Leiden mutation (FVL) after the Dutch city where they made their discovery in.^10^ This nucleotide replacement happened to be in the codon for the amino acid residue arginine 506 (C**G**A) normally present in the factor V molecule, creating a new codon (C**A**A) which is translated as glutamine. In order to inactivate FV, APC needs to recognize arginine at position 506 of the FV molecule (Figure 2). Because of the amino acid change in FVL, APC can no longer inactivate FV efficiently, but FV retains its coagulation capabilities and therefore carriers of FVL develop hypercoagulability which may clinically manifest as VTE episodes. Later studies showed that people with FVL were at higher risk of developing VTE (10-fold in heterozygous carriers and 30 to 140-fold in homozygous carriers).^9^–^16^ In addition, most homozygotes for FVL were reported to get at least one VTE event in their life time.^17^,^18^ This explains the great clinical and scientific consideration this mutation had appealed and the hundreds of studies conducted on its prevalence and risk for developing VTE in almost every part of the world.

## World Distribution of Factor V Leiden:

Since its discovery, several studies were conducted to determine the prevalence of FVL mutation in normal subjects and in patients with VTE, as well as to measure the risk value of this mutation in developing VTE. First reports appeared in Europe, which concentrated on populations of Caucasian origin. They found FVL to be present in a quite high percentage of patients with VTE (15–65%) and healthy subjects (1–15%). Similar results were obtained when Caucasians where studied in non-European countries like USA, Australia and Israel. Table 1 gives examples of studies on FVL in European and non-European Caucasians.^2^,^3^,^10^,^12^,^14^–^16^,^19^–^64^ However, when studies where extended to other ethnic groups, FVL was surprisingly found to be very rare in Africans, South-East Asians, Chinese, Japanese, American Indians, Greenland Eskimos and Aboriginals of Australia (Table 2).^21^,^58^,^61^,^65^–^78^ These findings suggested that FVL might have occurred as a single event sometime in the distant past in a common European Caucasian ancestor, whose offspring are the present time Caucasian carriers of this mutation living in Europe and other countries. This assumption was later strengthened by molecular studies that reported FVL to be always associated with one haplotype of single nucleotides polymorphisms (SNPs), as will be discussed later. Later on, studies were conducted on Arabs and populations living in the Middle East and North Africa (The MENA region), as summarized in Table 3.^31^,^63^,^79^–^133^ These studies showed a high prevalence of FVL in these populations, who are not usually classified as Caucasians. However, the MENA region is geographically very close to Europe and had witnessed a lot of human movement from and to Europe, and hence such populations are expected to have some Caucasian genes in their DNA. Therefore, the presence of FVL in Arabs and North African populations should not be a surprising upshot.

## Factor V Leiden in the Mediterranean Region:

**Figure 3.**
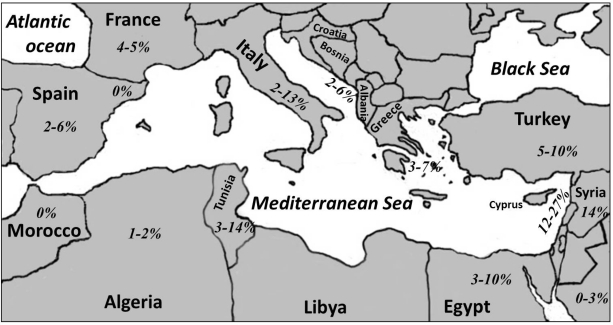
Map of the Mediterranean Sea and its countries showing the prevalence of FVL in healthy populations living there.

Currently, there are 20 countries that have seacoasts on the Mediterranean Sea and therefore are called the Mediterranean countries; 5 are in North Africa, 4 in West Asia and 11 in South Europe. Figure 3 gives a map of the Mediterranean Sea and its countries, showing the prevalence of FVL in these countries which are based on the studies listed in tables 1 **and** 3. No data could be retrieved from the literature on prevalence of FVL in Libya, Malta, Bosnia or Montenegro. However, one study reported FVL in Yugoslav people which included patients and healthy subjects from all over the former Yugoslavia, and possibly some of their subjects were from Bosnia and Montenegro.^48^ There were no reports from Albania itself, but one study was conducted in Kosovo, the people of which are considered as Albanians.^53^ In table 1, the prevalence of FVL in the people of the Basques was put separately, although not being a separate country, because of the unique rarity of FVL in these people.^42^,^43^ This has an important issue which will be discussed later. In Israel, the population consists of a mixture of Palestinian Arabs and other immigrants from different parts of the world, largely being of European Caucasian origin. Therefore, the results there were split between tables 1 **and** 3 according to the origin of the studied populations.

One may notice that the prevalence of FVL is present in its maximum peak in this part of the World. In addition, there is generally an Eas-to-West decline in prevalence of FVL in these countries. This observation was also noticed by Lucotte et al (2001) who also observed a South-to-North decline of these values in Europe, only when southwestern populations were excluded.^134^ These observations raised discussions in the literature on the exact place where FVL has first appeared.

## Has Factor V Leiden originated in the Eastern Mediterranean?

As was mentioned before, the first reports on the prevalence of FVL found high prevalence of FVL in European Caucasians, while the prevalence was almost zero in other ethnic groups. In addition, studies showed an association of FVL with one haplotype in all carriers of the mutation. Therefore, scientists got a perception that FVL has occurred once in the past time in one European Caucasian person. Anthropology proposes that Caucasoid populations who settled in Europe were diverted from Mongoloid populations (who moved to East Asia) around 32 thousands of years ago; therefore FVL should have appeared sometime earlier than 32,000 years ago.^2^,^4^,^11^,^135^–^137^ It was suggested that the mutation occurred in Europe first, and then spread to other parts of the world. However, the observed highest prevalence of FVL in Eastern Mediterranean countries have raised speculations that FVL might have occurred somewhere there and then spread to Europe.^43^,^99^,^100^,^134^,^138^,^139^ The author of this paper has found FVL to be associated with one haplotype in 67 Arabs from Eastern Mediterranean region, which was the same haplotype found in European carriers of the mutation, giving another confirmation that FVL occurred as a single event in the past even in Arabs. 140 Castoldi et al (1997) suggested that FVL probably occurred outside Europe.^138^ The rarity of FVL in the French and Spanish Basque populations, which are thought to be the oldest ethnic groups in Europe of Paleolithic origin, has also suggested FVL to occur outside Europe first.^42^,^43^ Lucotte et al (2001) proposed that FVL expanded in Europe during the Neolithic period, from a probable Anatolian center of origin in Turkey, which has occurred around 10,000 years ago.^134^ This may explain the highest prevalence of FVL in East Mediterranean countries, and that the prevalence decreases when radiating away from this region towards Europe or other parts of the world. Still, more genetic and molecular studies may be needed to detect certain genetic loci or markers that may help in following the movement of carriers of FVL in the Mediterranean region to definitely determine the exact location where FVL might have occurred first.

## Conclusions:

Molecular and epidemiological studies provide evidences that FVL should have occurred as a single event in the past. The Mediterranean region has the highest prevalence of FVL in the world. This suggests it as the area where this mutation has arisen, possibly 10,000 years ago, and then it was spread to other parts of the world.

## Figures and Tables

**Table 1.** Prevalence of FVL in Caucasian patients with VTE and normal subjects living in European and non-European countries. European countries on the Mediterranean Sea are shown.

	***Country***	***VTE patients (%)***	***Normal Population (%)***	***Reference***
***European Caucasians Non-Mediterranean***	UK	----	1.74–5.6	*19, 20*
	Sweden	41.5–50	7.5–11.4	*14, 15, 19*
	Poland	----	5	*21*
	Netherlands	21	2	*10, 20*
	Germany	30	7.1–12	*22, 23*
	Belgium	22	3.3	*24*
	Slovakia	29.5–37.0	4	*25, 26*
	Austria	26	----	*27*
	Hungary	44	6.9	*28, 29*
	Serbia	29.9	5.8	*30*
	Azerbaijan	----	14	*31*

***European Caucasians Mediterranean***	Spain	9.2–26.3	1.6–5.8	*32–37*
	France	9–18	3.5–5.0	*38–41*
	French/Spanish Basques	----	0–0.7	*42, 43*
	Italy	9.0–42.8	2–13.1	*12, 44–47*
	Yugoslavia	15.5	4.0	*48*
	Slovenia	12.9	6.3	*49*
	Croatia	21.0–28.2	2.4–4.0	*50–52*
	Albania/Kosovo	----	3.4	*53*
	Greece	16.2–31.9	2.5–7.0	*2, 54–57*

***Non European Caucasians***	USA	8.6	3.2–6.0	*3, 16, 58*
	Australia	----	4–10.2	*59–62*
	Israel	----	4.3	*63*
	Brazil	20	2	*64*

**Table 2.** Prevalence of FVL in non-Caucasian patients with VTE and normal subjects in different parts of the world.

	***Country/Ethnic groups***	***VTE patients (%)***	***Normal Population (%)***	***Reference***
***Asians***	Japan	0	0	*65–68*
	Korea	0	----	*69*
	China	0	0	*70, 71*
	Indonesia	----	0	*70*
	Malaysia	0.5	----	*72*
	Singapore	5	----	*72*
	India	3	1.3	*21, 73*
	Pakistan	1.25	----	*74*
	USA	----	0	*75*

***Africans/Black***	Ethiopia		0	*70,76*
	USA	1.4	0.9	*58, 75*
	Sub-Sahara	----	0	*70*
	Ecuador	----	0	*70*
	Venezuela	----	4.4	*77*

***Amerindians***	Ecuador	----	0	*70*
	Venezuela	----	1.25	*77*
	USA	----	0	*75*

***Eskimos***	Greenland	----	0	*78*

***Indigenous Australians***	Australia	----	0	*61*

**Table 3.** Prevalence of FVL in patients with VTE and normal subjects in Arabs and non-Arabs living in different Middle-Eastern and North African countries. Countries on the Mediterranean Sea are shown.

	***Country/Ethnic groups***	***VTE patients (%)***	***Normal Population (%)***	***Reference***
***North Africa Mediterranean***	Morocco	----	0	*79–80*
	Algeria	13.8	1.3–2.0	*81, 82*
	Tunisia	20.3–24.6	3.0–13.6	*83–92*
	Egypt	30	2.5–10.2	*93–97*

***Middle East Mediterranean***	Palestine (inside & outside Israel)	----	11.7–27.2	*63, 97, 98*
	Lebanon	9.9–70.6	13.6–18.7	*89, 92, 99–106*
	Syria	----	13.6	*97, 99*
	Turkey	21–30.8	4.6–9.8	*31, 107,108–115*
	Cyprus	----	13.4	*116*

***Middle East Non-Mediterranean***	Jordan	23.9–25.7	10.5–27.2	*97, 117–122*
	Iraq	----	7.0	*123*
	Kuwait	15.8	2–4.5	*97, 124*
	Saudi Arabia	----	0–2.5	*89, 123, 125*
	Bahrain	52	3.1–14.7	*89, 126*
	Oman	0	0	*127*
	Yemen	----	0	*63*
	Iran	11.4	2.0–10.6	*123, 128–133*
